# Bioethics and the use of social media for medical crowdfunding

**DOI:** 10.1186/s12910-020-00521-2

**Published:** 2020-10-06

**Authors:** Brenda Zanele Kubheka

**Affiliations:** 1 School of Public Health, Faculty of Health Sciences, University of Witwatersrand, Johannesburg, South Africa; 2 Health IQ Consulting, Johannesburg, South Africa

**Keywords:** Medical crowdfunding, Justice, Social media, Vulnerability, Privacy, Ethics, Undue influence

## Abstract

**Background:**

Social media has globalised compassion enabling requests for donations to spread beyond geographical boundaries. The use of social media for medical crowdfunding links people with unmet healthcare needs to charitable donors. There is no doubt that fundraising campaigns using such platforms facilitates access to financial resources to the benefit of patients and their caregivers.

**Main text:**

This paper reports on a critical review of the published literature and information from other online resources discussing medical crowdfunding and the related ethical questions. The review highlighted the benefits of crowdfunding as well as the under-exploration of the risk of having patients’ desires and human rights undermined during online fundraising campaigns. Majority of these campaigns get initiated on behalf of the patients, especially the very sick and dependant. The ethical questions raised relate to the voluntariness of informed consent and the possibility of patients being used as a means to an end. Vulnerability of patients may expose them to coercion, undue influence, manipulation, and violation of their human rights. The success of these campaigns is influenced by the digital skills, pre-existing social networks and, the emotional potency. Healthcare is a public good, and online market forces should not determine access to essential health services. The benefits of crowdfunding cannot be subverted, but it can perpetuate unintended injustices, especially those arising from socio-economic factors.

**Conclusions:**

Policymakers ought to monitor the utilisation of crowdfunding sites to identify policy failures and unmet essential health care needs responsible for driving individuals to use these platforms. The upholding of human rights and the fundamental respect of the individual’s wishes is a moral imperative. The need for an ethics framework to guide different stakeholders during medical crowdfunding needs further examination.

## Background

The use of social media as a communication and a transactional tool has grown significantly in recent years. Social media presents new opportunities, including digitised and globalised charity activities like crowdfunding. Crowdfunding is defined as the raising of funds from a large pool of donors for specific initiatives through an online public appeal [1–3]. Medical crowdfunding is the practice of using websites and social media platforms to raise funds from donors to cover expenses directly or indirectly related to medical care [2, 4, 5]. The use of social media for crowdfunding raises moral questions, especially for donations to cover expenses related to medical treatment.

In healthcare, crowdfunding is used for fundraising to meet different needs ranging from medical care through to biomedical research. This also includes access to an experimental treatment that is not evidence-based, raising medico-legal concerns [1–3, 6]. The benefits of crowdfunding campaigns range from facilitating access to care, debt avoidance, and allowing family members to spend more time with sick loved ones [2]. Notably, some patients resort to crowdfunding for direct health care expenses because of shortfalls in medical insurance cover; inadequate public health funding and desperation caused by financial strain from life-threatening medical conditions [ [2, 6]].The drive for crowdfunding may be compounded by system failures resulting in barriers to accessing care and limited availability of services, to name a few. Medical crowdfunding is a symptom of a problem, rather than a solution. It may be a sign of a failing system in countries with universal health insurance that is limited to essential medical services like Canada; and countries without it like the US [2, 7]. Snyder calls for policymakers to identify and address injustices motivating people to use these platforms to fund essential medical services [2].

Interestingly, Perry cites crowdfunding to have been traced as far back as in the 1800s, commenting that it was “a backbone of the American political system since politicians started kissing babies^.”^ [3]. This observation contradicts the notion of crowdfunding being a new-fangled phenomenon limited to online platforms. Politicians have sourced small monetary donations from large pools of people, which is different from the more efficient online crowdfunding, considered a new phenomenon [3, 5]. In the African context, sharing and caring are some of the characteristics of Ubuntu, an African philosophy premised on communitarianism, making benefiting from crowd-sourced resources like food and money not so foreign a concept. In this context however, contributors are usually limited to people known to the beneficiary, like family, friends, and neighbours.

Social media platforms facilitate borderless online communication allowing information shared on these to attain global footprints. Undoubtedly, social media’s popularity has facilitated the visibility and flourishing of crowdfunding platforms. These campaigns have different outcomes, and the most successful one is GoFundMe’s fundraising of $2 million for a young girl suffering from a rare neurological disorder [6].

## Main text

### Crowdfunding landscape

Crowdfunding campaigns are used for raising funds for various projects. Campaigns are initiated by or on behalf of beneficiaries, which may be individuals or organisations. There are two types of crowdfunding; donation-based used for charity and personal treatment without an expectation of a reward for the money received. Alternately, reward-based is when donors are promised a product or a service in return for their financial contributions. The latter is mainly seen in research projects [5] and business ventures. The Internet and social media have broken down international barriers, thus facilitating interconnectedness and globalisation of compassion [8]. A knowledge gap exists in this specific area because of the lack of oversight and reporting from these platforms [5, 6]. Undoubtedly, there are potential benefits to be reaped from trend analysis and the scrutiny of factors influencing the use of such platforms by patients and caregivers. Harvesting such information could assist policymakers in identifying society’s unmet needs [6].

Approximately 62% of individual bankruptcy filings recorded in 2007 in the US were a result of medical expenses, even though most of these people had insurance. In the same period, crowdfunding by individuals in America is attributed to have saved between 114 and 136 bankruptcy cases per quarter [5–7]. Critical to this paper is the review citing the success of crowdfunding campaigns to be higher in countries with low public health coverage, suggesting a substitution effect between crowdfunding and public health insurance [5]. Interestingly, a study conducted in Canada identified requests for donations to range from funding the actual treatment, time off work, hospital parking fares, travel, and other living expenses [9].

### Ethico-legal concerns

Caregivers normally initiate crowdfunding campaigns. Health professionals have no legal obligation to validate the accuracy of the information disseminated in these appeals. Therefore, sharing of inaccurate information poses moral dilemmas for professionals stemming from conflicting ethical obligations towards patients and the society. Furthermore, clinical information may be exaggerated for emotional potency, and enhanced financial incentive [6]. Some concerns also arise from the exploitation of vulnerable patients who may be used by treatment centres or clinicians to access funding for dubious research [5, 6]. Other concerns are related to fraudulent campaigns, as evidenced by a Canadian woman who faked a cancer diagnosis and raised thousands of dollars [7]. There are different views about the moral responsibilities of campaign initiators towards donors and society at large; this requires further scrutiny [1, 2, 7].

#### Crowdfunding perpetuating inequality

There is no doubt that crowdfunding has positive benefits, conversely however, such strategies may indirectly facilitate the commodification of healthcare, which ought to be a public good. Crowdfunding creates an opportunity for market forces to determine who gets funding and for what conditions [2, 5, 9]. This may lead to unintended consequences like undue influence on the allocation of resources and inequitable access to finance based on the social status of the patient or campaigner, and their existing social network [8, 9]. Emotionally appealing pictures, digital marketing skills, and frequent updates are some of the factors influencing the success of campaigns [1, 2]. Evidently, successful campaigns are seen in patients or caregivers with extensive pre-existing networks of people; donors tend to be from the same socio-economic class. Crowdfunding websites offer an option to share campaigns on social media platforms like Facebook, therefore, giving individuals with existing extensive social networks access to a broader audience of potential donors. People who are well connected are more likely to have successful campaigns, thus perpetuating inequality. In this way, donor decision-making is vulnerable to influence from social value instead of medical need [9] perpetuating unintended discrimination. More so in countries with significant socio-economic inequality, typically accentuated by a digital divide, further propagating injustices.

#### Coersion and undue influence

There is a significant body of knowledge on ethical issues related to paternalism and soft coercion by clinicians and lately, on undue influence by family members on care decisions of very sick patients. Clinicians are advised to assess the voluntariness and competence of terminally ill patients to ensure that manipulation, relational, and undue influence does not undermine the patient’s wishes [10–12]. The family’s wishes and priorities may be different from those of the patient. Ho asserts a significant role played by family and other intimate relationships in a patient’s wellbeing and recovery. Ho argues that families also play a role in preserving the patient’s identity and therefore, ought not to be viewed negatively [11]. Patients do not exist in isolation; cultural beliefs also influence decision making structures [11, 12]. These relationships may give rise to conflicts because of the competing interests of the family, versus the autonomy of the patient. These may be compounded the existing power dynamics in a family structure [11, 12]. Moral issues related to undue influence and manipulation of patients associated with online fundraising campaigns remain under-explored. Arguments can be made about the inability of the family to meet the financial means required to fund medical care and justify the need to avoid becoming destitute as a result of the illness. Vitally, patients ought not to be treated as a means to an end. They must have room to exercise their agency over determining their ends.

#### For-profit interests and commodification of healthcare

The case of Charlie Gard was one such crowdfunding campaign which managed to raise more than $1,2 million within 6 months to fund experimental treatment for a rare medical condition. The doctors who examined and treated him had agreed that the specific therapy was not going to be effective in this case [9]. The incident raised concerns about the nature of the doctor-patient relationship; the doctor’s obligation to his patient, and the obligation to practice evidence-based medicine. Prominent figures like Donald Trump and Pope Francis publicly proclaimed their support for Charlie’s campaign [13]. The influence of celebrities’ social capital cannot be underestimated as it facilitates peer-to-peer fundraising and the unleashing of “Star Power” to benefit the campaign. In this case, the media played a significant role, interviewing the parents on television and sharing their pictures on various online platforms. GoFundMe donated $10,000 to Charlie’s campaign hosted on their platform but had refused to waive platform fees on a campaign supporting victims of a Somali drought in 2017, raising ethical disquiet. Of concern, is a gatekeeper role conferred on these platforms in deciding which campaigns are promoted, thus indirectly determining who gains access to care [9]. Similarly, it seems that crowdfunding websites are passive platform providers to campaigners without corresponding moral accountability [14].

#### Vulnerability and informed consent

Direct marketing to consumers of unapproved therapies like stem-cell treatments for various medical conditions, including cancer, has increased in recent years, particularly in the US, Australia, and Japan. Adverts for experimental and dubious treatment prey on the vulnerability of patients and their loved ones. Such access to experimental treatments raises patient safety concerns regarding beneficence and the adverse effects of unproven treatment modalities. Ominously, patient safety may be the least concern because of false promises made by the treatment providers combined with vulnerability stemming from the desperate need to live longer. Patients may feel pressured to enrol in clinical trials and subject themselves to dubious treatments. Some of these questionable clinical trial results may even be published in academic journals. Of concern, is the 2016 report that documented 351 US companies selling alleged stem-cell treatments direct to consumers [15–17].

A life-threatening diagnosis is considered a constraining situation, which may impact voluntariness of informed consent, especially in patients that are entirely dependent on their caregivers. Constraining conditions are non-intentional, coercion-like situations that cause a person to feel controlled by constraints such as severe illness or poor access to health services; circumstances not due to the design of another person [17, 18]. Majority of patients are either too young or too sick to start these campaigns, thus placing them in a vulnerable position. Decisions are made on their behalf by caregivers or family members who decide on the extent of the information to be shared to raise funding [9]. Patients may feel compelled to agree to have pictures taken, and private information shared on social media platforms because of a state of powerlessness caused by the need for funding [17]. They may even end up subordinating their rights to privacy in a bid to please caregivers.

Vulnerability is intrinsic to the human condition. It is experienced differently by individuals based on access to resources, and there are links to sociability and dependency on others. Vulnerability remains under-explored within medical research ethics and similarly in medical crowdfunding. Rodriguez argues that this is a result of ethics being a rationalist field; hence it ignores feelings. Meanwhile, human beings have an inherent vulnerability. Sick and dependent individuals are particularly susceptibility to harm caused by exploitation with their autonomy not being upheld, irrespective of intention [19]. Caregivers ought to avoid assuming a paternalistic role for patients who have the capacity to make decisions for themselves. The motive of the crowdfunding campaign must uphold the patient’s wishes and beliefs. Beneficence should outweigh disadvantages to the patient, which includes avoiding unintended harm [10]. Conversely, this raises the question of whether it is acceptable to harm the family finances for the sole reason of preserving an individual’s autonomy should they not consent to sharing of personal information for fundraising purposes. The success of crowdfunding campaigns is not guaranteed, and it ought not to be done at all costs to the patients. Patients with decisional capacity have a right to withdraw their consent at any time.

#### Privacy and the responsibility to others including future generations

Crowdfunding platforms are for-profit, and the relationship between them and the campaigners is transactional and may promote commodification of healthcare. In Charlie’s case, GoFundMe charged 5% for the use of the platform, 2,5% processing fee, and $0.20 per donation, meaning that they generated a six-figure fee from this campaign. These platforms and other websites offer advice on how to create successful crusades, and this includes using videos, sharing sensitive information, and providing regular updates stimulating emotional currency. Not surprisingly, campaigns characterised by excessive sharing of personal information are the most successful. This level of information sharing serves as an incentive to sensationalise stories, raising privacy concerns [1, 2, 6, 9]. Every human being has a right to enjoy freedom from unauthorised intrusion and personal information disclosures. Privacy is grounded on the principles of respect for persons and autonomy. Human beings are inherently dignity deserving with the right to self-determination and not to be treated as the means to an end.

Information shared online leaves permanent footprints, and these may have future ramifications. For instance, disclosure of personal information related to certain types of cancers and rare genetic diseases might facilitate dissemination of genetic information about other family members without their informed consent [20].At the same time, the benefits of sharing such information cannot be discounted as it may facilitate support from relevant groupings and other patients. It may also ease the identification of target patients should discovery of a cure be made. Despite this, individuals might not want to have a digital identity. Caregivers can undermine personal wishes, or make a patient feel softly coerced to having their personal information shared online for fundraising purposes. Illegal invasion of privacy that threatens informed consent and the patient’s wishes, compromises the right to self-determination and is therefore unethical [17]. The right to freedom of expression by caregivers comes with a corresponding responsibility to prevent avoidable harm and violation of other people’s rights. Therefore, the vulnerability of patients requires express consent as well as legal justification for sharing sensitive information online.

These concerns are relevant even when patients have the capacity to make decisions. Voluntariness may still be compromised in cases where a patient feels indebted, burdensome, or dependent on a caregiver. The patient can agree to have information shared online due to a sense of powerlessness and vulnerability. Caregivers or others setting up a crowdfunding campaign have an obligation to protect the patient. Therefore, they must be responsible for advising on both the benefits and risks of sharing private information online to raise funding for medical treatment.

Crowdfunding fails to address the fundamental questions of underfunding for specific medical conditions. It detracts attention from the structural problems related to healthcare funding and promotes individualised charity in highly competitive social media platforms [21].This was seen in a recent case involving a 31-year-old South African (SA) woman diagnosed with a brain tumour after suffering multiple seizures during a visit to the UK. Consultations between the SA and British doctors concluded that flying back to SA for surgery was going to be risky. Challengingly, the patient’s medical scheme only agreed to fund a portion of the treatment costs abroad. This led her husband, a marketing manager for a multinational pharmaceutical company, to turn to the public, raising the required shortfall of R400 000.00 within 2 days using social media crowdfunding [22].

## Conclusion

Medical crowdfunding provides a link between people needing donations to fund medical care and altruistic donors. There is no doubt that it can bridge gaps between treatment needs and the required funding. It does however present ethical challenges for policymakers, vulnerable patients, and society, hence the need for moral scrutiny. Crowdfunding has unintended consequences; allowing individuals with excellent digital communication skills and extensive networks to have an unfair advantage compared to the rest of society. Therefore, policymakers ought to monitor the use of these platforms to identify unmet needs and prevent diversion of attention from policy failures. There is a need to explore the level of manipulation and exploitation of vulnerable patients without discounting the value brought by these platforms. More research is required to examine moral issues related to undue influence and manipulation faced by vulnerable patients. The development of an ethics framework ought to be explored to facilitate respect for persons and protect human rights during these online fundraising campaigns.

## Data Availability

not applicable.
